# Signaling network inhibition by selected traditional Chinese medicine preparations: a novel multi-target paradigm for colorectal cancer therapy

**DOI:** 10.3389/fphar.2026.1719306

**Published:** 2026-02-09

**Authors:** Mingming Zhang, Haoyang Wang, Junying Pan, Yuzhe Jin, Zengde Tan

**Affiliations:** 1 Tuina Department, The First Affiliated Hospital of Heilongjiang University of Chinese Medicine, Harbin, China; 2 Graduate School of Heilongjiang University of Traditional Chinese Medicine, Harbin, China

**Keywords:** colorectal cancer, crosstalk, multi-target therapy, signaling pathway, traditional Chinese medicine therapy

## Abstract

Colorectal cancer (CRC) remains a significant global health burden, with drug resistance, recurrence, and metastasis posing major therapeutic challenges. Traditional Chinese Medicine (TCM), employing a holistic “multi-component, multi-target, multi-pathway” strategy, offers a promising complementary approach. This review systematically summarizes recent advances in how TCM monomers and formulations regulate key CRC signaling pathways—including PI3K/Akt/mTOR, MAPK, Wnt/β-catenin, Hedgehog, and NF-κB—to inhibit tumor proliferation, promote apoptosis, suppress metastasis, and remodel the tumor microenvironment. Emphasis is placed on TCM’s unique modulation of pathway crosstalk, providing new insights into its ability to reverse drug resistance and enhance chemosensitivity. The review further proposes and discusses the future translational potential of TCM-based “signaling network inhibitors” as an innovative strategy in CRC management.

## 1 Introduction

Colorectal cancer (CRC) is one of the most common malignant tumors of the digestive tract worldwide, with its incidence and mortality consistently ranking high on the global cancer list (Murphy and Zaki, 2024). According to the latest global cancer statistics, the annual number of new CRC cases has exceeded 1.9 million, and the number of deaths attributed to it is close to 1 million, posing a substantial global health burden (Murphy and Zaki, 2024). Its pathogenesis is closely associated with age, genetic factors, Westernized dietary patterns (such as high-fat, low-fiber diets), obesity, smoking, and chronic intestinal inflammation, among others (Sninsky et al., 2022).

Currently, the standard clinical treatment for CRC primarily involves surgical resection, supplemented by chemotherapy, radiotherapy, targeted therapy, and immunotherapy (Shin et al., 2023). However, these mainstream therapies, especially chemotherapy and targeted therapies focusing on single molecular targets, are often accompanied by severe toxic side effects, primary or acquired drug resistance, and a high risk of recurrence after treatment (Biller and Schrag, 2021). Fundamentally, tumors are not driven by isolated, linear signaling pathways but rather constitute a complex, dynamic, and highly redundant signaling network involving core pathways such as PI3K/Akt/mTOR, MAPK, Wnt/β-catenin, Hedgehog, and NF-κB. Tumor cells exploit the complexity and compensatory mechanisms of this network; when one pathway is inhibited by drugs, they can often rapidly compensate through other parallel or interacting (crosstalk) pathways, thereby evading therapeutic pressure and leading to drug resistance (Sun et al., 2024; Zheng et al., 2025; Wang et al., 2022). Consequently, “sniper-style” therapies targeting single nodes have inherent limitations in addressing the heterogeneity and adaptability of tumor signaling networks.

In this context, Traditional Chinese Medicine (TCM), with its holistic regulatory philosophy of “multi-component, multi-target, multi-pathway” intervention, offers a unique perspective and strategy to overcome the above challenges (Zheng et al., 2025). Chinese herbal monomers and compound prescriptions are rich in various bioactive components. Their action is not a simple superposition of targets but involves systemic intervention in multiple key nodes and their crosstalk within the tumor signaling network through synergy and antagonism. This mode of action can simultaneously influence multiple malignant biological processes, including cell proliferation, apoptosis, metabolism, invasion, metastasis, and tumor microenvironment remodeling (Rao et al., 2025). Its scientific advantage lies in potentially weakening multiple driving pathways simultaneously at the network level and preemptively blocking or interfering with the transmission of compensatory activation signals, thereby more effectively inhibiting tumor growth and delaying or reversing the emergence of drug resistance (Sun et al., 2024; Zheng et al., 2025; Wang et al., 2022). This essentially represents a strategy of “signaling network inhibitors,” forming a distinct complement to the traditional single-target approach.

This review aims to systematically elucidate the latest research progress on how Chinese herbal monomers and compounds inhibit tumor progression by regulating the aforementioned core signaling pathways and their complex interactive networks in CRC. The article will not only update specific research examples within each pathway but will also place a special emphasis on dedicated analyses exploring how TCM intervenes in crosstalk nodes between key pathways, such as “Wnt/β-catenin and Hedgehog,” “PI3K/Akt and NF-κB,” and “MAPK and PI3K/Akt,” revealing the deeper mechanisms of their integrated regulation and synergistic tumor suppression. By breaking down the isolated research barriers between different pathways and adopting a more systemic and holistic perspective to uncover the pharmacological action network of Chinese medicine, this review hopes to provide a solid theoretical foundation and clear research direction for the future development of novel, efficient, and low-toxicity “network therapies” against CRC based on TCM principles.

## 2 Literature search

To ensure the comprehensiveness and objectivity of this review, we followed a systematic literature search and screening process. The search was conducted in June 2025, aiming to cover recent research advancements up to that time.

Search Databases: The primary electronic databases searched include: English literature via PubMed, Web of Science Core Collection, and Embase; Chinese literature via China National Knowledge Infrastructure (CNKI), Wanfang Data Knowledge Service Platform, and VIP Chinese Journal Service Platform.

Search Time Frame: The search was limited to literature published from January 2019 to June 2025 to focus on research advances over the past 5 years.

Search Keywords: A combination of subject headings and free-text terms was employed. The core structure of the search strategy is as follows: (colorectal cancer OR colon cancer OR rectal cancer) AND (traditional Chinese medicine OR Chinese herbal medicine OR herbal medicine OR phytotherapy) AND (signaling pathway OR PI3K/Akt OR MAPK OR Wnt OR Hedgehog OR NF-κB OR crosstalk).

### 2.1 Data extraction and quality assessment

In addition to the mechanistic findings, data regarding the characterization of TCM materials were extracted, including (where available): the botanical source (Latin name, plant part used), extraction method and solvent, standardization markers (if any, e.g., content of a specific compound), and dosage/form used in the experiments. It is noted that a significant portion of the reviewed studies, particularly earlier ones, lack comprehensive reporting of these parameters, which is acknowledged as a limitation affecting the reproducibility and comparative analysis of the evidence.

During data extraction, particular attention was paid to the characterization of the TCM materials under investigation. Where available, we documented key parameters including the botanical source (with Latin binomial nomenclature), specific plant part used, extraction method and solvent, chemical standardization details (e.g., HPLC fingerprint, content of a primary bioactive marker), and the dosage form applied in the experimental models. However, it must be noted that a significant proportion of the reviewed studies, especially those published earlier, lack comprehensive reporting of these essential parameters. This variability and incompleteness in material description represent a critical limitation that affects the reproducibility and comparative validity of the findings across different studies, and is acknowledged as a major challenge in the field.

## 3 Multitarget signaling pathways with interconnected regulatory networks

### 3.1 PI3K/Akt/mTOR signaling pathway

The PI3K/Akt/mTOR signaling pathway plays a central regulatory role in the development and progression of CRC. This pathway, activated by extracellular signals such as growth factors and cytokines, regulates key biological processes including cell proliferation, survival, metabolism, and apoptosis (Glaviano et al., 2023). In CRC, aberrant activation of this pathway—often due to PTEN loss, PI3K mutations, or Akt overexpression—promotes uncontrolled tumor cell proliferation, inhibits apoptosis, and contributes to metabolic reprogramming and tumor microenvironment remodeling, making it an important target for TCM intervention (Yu et al., 2022).

Multiple studies have demonstrated that TCM monomers and compound formulations can inhibit CRC progression by modulating the PI3K/Akt/mTOR pathway. For example, Feng Yewen et al. found that cryptotanshinone upregulates PTEN expression, suppresses PI3K/Akt pathway activity, and downregulates cell cycle proteins such as Cyclin D1 and CDK4/6, thereby inhibiting the proliferation of Caco-2 cells (Feng et al., 2024). Wen Luwen et al. reported that Trametes robiniophila extract downregulates P85α and p-AKT expression, increases the BAX/Bcl-2 ratio, and activates Caspase-3/9, inducing apoptosis in HCT8 and HT29 cells (Wen et al., 2023). Further, Zhang Shuying et al. revealed that pachymic acid inhibits the PI3K/Akt/mTOR pathway, reduces the expression of key glycolytic enzymes HK2, GLUT1, and LDHA, and thereby suppresses glucose metabolism and promotes apoptosis in CRC cells (Zhang S. Y. et al., 2024).

**FIGURE 1:**
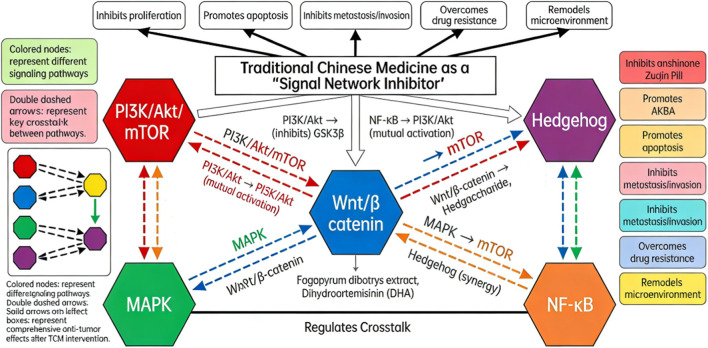
Signal network diagram of colorectal cancer regulated by traditional Chinese medicine.

Moreover, TCM compound formulations demonstrate multi-target advantages in regulating the tumor immune microenvironment and metabolic reprogramming. Nong Feifei et al. discovered that Zuojin Pill downregulates TIPE1 expression, inhibits the PI3K/Akt pathway, and reverses the polarization of tumor-associated macrophages toward the M2 phenotype, thereby suppressing the proliferation and migration of CT26 cells (Nong et al., 2025). Liang Yuwei et al. confirmed that Anzheng Kangliu Formula dose-dependently inhibits the expression of p-PI3K, p-Akt, p-mTOR, HK2, and LDHA in SW620 cells and xenograft tumors, reducing glucose consumption and lactate production—suggesting its anti-CRC effect via suppression of the PI3K/Akt/mTOR pathway and regulation of glycolysis (Liang et al., 2025). Collectively, these studies illustrate that TCM, through its multi-component, multi-target regulation of the PI3K/Akt/mTOR pathway and its crosstalk mechanisms, holds promising potential in the treatment of colorectal cancer, show as Figure 1.

### 3.2 MAPK signaling pathway

The MAPK signaling pathway, a critical intracellular signal transduction network, plays a central role in the development and progression of CRC by regulating key biological processes such as cell proliferation, apoptosis, autophagy, migration, and invasion. This pathway primarily includes the ERK, JNK, and p38 MAPK branches, which maintain cellular homeostasis under physiological conditions but, when aberrantly activated in CRC (e.g., through elevated phosphorylation levels), promote unlimited proliferation, inhibit apoptosis, and enhance metastatic potential, making it an important therapeutic target (Park, 2023). TCM and its active components, leveraging their multi-component and multi-target advantages, exert anti-CRC effects by precisely modulating the MAPK signaling pathway and its crosstalk with other pathways, offering new strategies for developing low-toxicity, high-efficacy antitumor therapies.

Xu Guangya et al. found that an extract of Fagopyrum dibotrys significantly inhibited proliferation, reduced viability, and induced apoptosis in human CRC HCT-116 cells, while upregulating autophagy-related proteins Beclin1 and LC3B and downregulating p62. Further mechanistic studies revealed that Fagopyrum dibotrys induces reactive oxygen species (ROS) generation, activating phosphorylation of ERK and JNK in the MAPK pathway (increased p-ERK and p-JNK expression). The ROS scavenger NAC reversed these effects, indicating that the antitumor action of Fagopyrum dibotrys depends on synergistic regulation of the ROS/MAPK signaling pathway (Xu G. Y. et al., 2024).

Wang Xiaoshan et al. investigated the combined application of the TCM compound Xihuang Pill with the targeted drug osimertinib, finding that Xihuang Pill enhanced osimertinib’s cytotoxic effects on HCT8 and DLD-1 CRC cells and patient-derived organoids. This synergy was linked to inhibition of the JNK/p38 MAPK/NF-κB pathway, with the combination significantly reducing p-JNK, p38 MAPK, and NF-κB protein expression, as well as downregulating anti-ferroptosis proteins p-Nrf2 and FSP1. Positive scores in synergy models (HSA, Loewe, ZIP) confirmed that Xihuang Pill enhances osimertinib’s antitumor activity through crosstalk between the MAPK and ferroptosis pathways (Wang et al., 2025).

Studies by Yu et al. (2024) and Wu et al. (2023) jointly elucidated the mechanism by which TCM compounds regulate CRC cell apoptosis via the p38 MAPK/p53 pathway. Serum containing Diyu Huaihua Decoction (15%, 20% doses) significantly inhibited HCT116 cell proliferation, induced apoptotic morphology (nuclear shrinkage and fragmentation), and upregulated Bax/Bcl-2 ratio, Cleaved caspase-3, p-p38 MAPK/p38 MAPK, and p53 protein expression, while downregulating MDM2. The p38 MAPK inhibitor TAK-715 reversed these effects (Yu et al., 2024). Similarly, serum containing Zishen Gusui Decoction (20% dose) promoted HCT-116 cell apoptosis by activating the p38 MAPK/p53 pathway, increasing p-p38 MAPK, p53, and Bax expression, and decreasing MDM2 and Bcl-2 expression. The p38 MAPK inhibitor SB203580 attenuated this pro-apoptotic effect (Wu et al., 2023), indicating the p38 MAPK/p53 pathway as a key target for TCM compound-induced apoptosis in CRC.

Additionally, Xu J. R. et al. (2024) discovered that dihydroartemisinin (DHA) exerts anti-CRC effects by modulating the MAPK/PI3K/Akt signaling pathway. DHA (95 μmol/L) significantly inhibited RKO cell proliferation and migration, arrested the cell cycle at the G_2_/M phase, induced intrinsic apoptosis (upregulating Cleaved caspase-3/Caspase-3, Cleaved caspase-9/Caspase-9, and Bax/Bcl-2 ratio), and reduced p-p38 MAPK, p-PI3K, and p-Akt protein expression. These findings indicate that DHA synergistically suppresses CRC progression by inhibiting crosstalk between the MAPK and PI3K/Akt pathways, show as Figure 1.

### 3.3 Wnt/β-catenin signaling pathway

The canonical Wnt/β-catenin signaling pathway serves as a core regulatory network in the initiation, progression, and metastasis of CRC, playing a dual role in maintaining cellular homeostasis and driving tumor development. Under physiological conditions, cytoplasmic β-catenin is phosphorylated and ubiquitinated for degradation by the APC/Axin/GSK3β destruction complex, maintaining low expression levels. In CRC, however, APC gene mutations and aberrant β-catenin phosphorylation sites disrupt the degradation complex, leading to β-catenin accumulation in the cytoplasm and its translocation into the nucleus. There, it forms a complex with TCF/LEF transcription factors, activating target genes such as CyclinD1 (cell cycle regulation), c-Myc (cell proliferation), and Snail (epithelial-mesenchymal transition, EMT), thereby driving abnormal cell proliferation, inhibiting apoptosis, and promoting tumor invasion and metastasis (Song et al., 2024). Additionally, this pathway contributes to CRC liver metastasis (occurring in approximately 50% of patients) and chemotherapy resistance by regulating multidrug resistance proteins (e.g., P-gp), migration-related genes (e.g., Fosl1, FOXS1), and histone-modifying enzymes (e.g., KDM5B, JMJD2C), making it a critical factor in disease progression and treatment failure (Zhang and Wang, 2020).

Active components of TCM can target key molecules of the Wnt/β-catenin pathway to exert anti-CRC effects. Cheng Wei et al. demonstrated that an extract from Taxus chinensis concentration-dependently inhibits HCT116 cell proliferation and increases the apoptosis rate to 34.42%. *In vivo*, the extract reduced xenograft tumor volume and downregulated Wnt and β-catenin mRNA as well as CyclinD1 protein, thereby blocking this pathway (Cheng et al., 2025). Deng Lan et al. found that the frankincense-derived component AKBA dose-dependently inhibits proliferation (inhibition rate >80%), migration (wound healing rate reduced to 20.17%), and invasion (Transwell membrane-penetrating cells decreased to 14.35). The mechanism involves downregulation of β-catenin and c-Myc expression, and the effect is enhanced when combined with the Wnt blocker DKK1, confirming its targeted action on the pathway (Deng et al., 2024).

TCM can also enhance chemotherapy sensitivity and intervene in crosstalk with migration -related pathways through this pathway. Wu Xiangpeng et al. showed that astragalus polysaccharides increase the sensitivity of HCT116 cells to 5-FU: combination treatment reduced colony formation by over 60%, increased the apoptosis rate threefold, and raised autophagosome numbers fourfold. Mechanistically, the polysaccharides downregulated Wnt3a and β-catenin expression while upregulating Bax, Cleaved Caspase-3, and autophagy-related proteins. These effects were reversed by a Wnt pathway agonist, indicating that astragalus polysaccharides promote apoptosis and autophagy by inhibiting the Wnt/β-catenin pathway (Wu et al., 2025).

In summary, the Wnt/β-catenin signaling pathway is central to CRC progression and a key target for multi-target TCM therapy. The studies reviewed demonstrate that active components from Taxus chinensis, frankincense, and astragalus can directly inhibit this pathway, effectively suppressing tumor cell proliferation and migration. Astragalus polysaccharides, by modulating this pathway, significantly enhance the chemosensitivity of cancer cells to 5-fluorouracil, primarily through the induction of apoptosis and autophagy. These findings underscore the unique advantage of TCM in treating CRC via multi-target regulation of signaling networks, show as Figure 1.

### 3.4 Hedgehog signaling pathway

The Hedgehog (Hh) signaling pathway, frequently aberrantly activated in CRC, serves as a key mechanism driving malignant progression. Dysregulation of this pathway promotes nuclear translocation of Gli transcription factors, leading to upregulation of target genes such as c-Myc and Cyclin D1, which collectively stimulate abnormal cell proliferation, inhibit apoptosis, and regulate EMT, angiogenesis, and chemotherapy resistance (Hu et al., 2024). Furthermore, the close interaction between the Hh pathway and gut microbiota dysbiosis or the chronic inflammatory microenvironment provides a strong rationale for multi-target interventions using TCM.

Several TCM monomers have been shown to directly target core components of the Hh pathway, thereby inhibiting CRC. Berberine downregulates the expression of SHH, SMO, and Gli1 while upregulating the negative regulator SUFU, effectively suppressing tumor growth both *in vitro* and *in vivo*. It also modulates the gut microbiota, achieving dual regulation of the “pathway–microbiota” axis (Sun et al., 2022). Toosendanin directly binds to the SHH protein, inhibiting its expression and pathway activity (Zhang et al., 2022). Ursolic acid has shown efficacy against the non-canonical Hh pathway (SMO-independent), broadening the scope of TCM-mediated pathway intervention (Chen et al., 2022).

TCM also exerts multi-dimensional effects by inhibiting tumor invasion, migration, and reversing drug resistance via the Hh pathway. Erianin inhibits the Hh pathway, thereby suppressing key downstream processes including EMT and angiogenesis, which collectively contribute to reduced metastatic potential of CRC cells (Zhang et al., 2023). In the context of chemotherapy resistance, ginsenoside Rg3 reverses abnormal activation of the Hh pathway in 5-fluorouracil-resistant cells. Combined with chemotherapy, it significantly enhances antitumor efficacy and suppresses EMT (Bu et al., 2024).

Additionally, TCM coordinates anti-inflammatory and antitumor effects through the Hh pathway. In a colitis-associated cancer (CAC) model, baicalin inhibited the Hh pathway, simultaneously reducing both tumor burden and levels of inflammatory factors, revealing a synergistic “anti-inflammatory–anticancer” mechanism (Lin et al., 2023).

In summary, TCM monomers precisely target different components of the Hh pathway and modulate the tumor microenvironment and chemotherapy response, offering a promising multi-dimensional strategy for the precise treatment of colorectal cancer, show as Figure 1.

### 3.5 NF-κB signaling pathway

During the initiation and progression of CRC, the NF-κB signaling pathway plays a critical role. Activated through both canonical and non-canonical pathways, it regulates multiple biological processes including tumor cell proliferation, apoptosis, inflammatory responses, migration, invasion, and immune escape (Cornice et al., 2024). The canonical pathway is primarily triggered by pro-inflammatory factors and pathogen-associated molecular patterns, leading to IKK complex activation, IκBα degradation, and nuclear translocation of the p65/p50 dimer, which initiates transcription of downstream target genes. The non-canonical pathway relies on NIK-mediated IKKα activation, promoting the processing of p100 to p52 and its nuclear translocation with RelB, thereby contributing to lymphocyte development and tumor microenvironment remodeling (Rong et al., 2022). Persistent activation of the NF-κB pathway not only promotes cell cycle progression and inhibits apoptosis but also upregulates inflammatory factors and matrix metalloproteinases, establishing a microenvironment conducive to tumor growth and metastasis (Campbell et al., 2024). Multiple studies have shown that monomers and active components of TCM exert anti-CRC effects by modulating the NF-κB pathway. For example: Astragalus polysaccharides inhibit VEGF and PD-L1 expression via the TLR4/MyD88/NF-κB pathway, thereby enhancing immune function (Xue, 2024); Ganoderma lucidum polysaccharides suppress the TLR4/MyD88/NF-κB pathway and downstream inflammatory factors in an AOM/DSS-induced mouse model (Guo et al., 2021); Resveratrol reverses EMT through the SIRT1–NF-κB axis, inhibiting cell migration and invasion (Miao, 2018); Baicalin and licochalcone A inhibit tumor proliferation and enhance T-cell activity by downregulating the TLR4/NF-κB and Ras/NF-κB pathways, respectively (Liu et al., 2021). Additionally, TCM components that promote blood circulation and resolve stasis—such as crocin and the natural cyclic peptide RA-VII—inhibit the TNF-α/NF-κB/VEGF pathway and the Akt/STAT3/NF-κB axis, respectively, exerting anti-angiogenic and anti-inflammatory effects (Lin et al., 2022). These findings highlight the potential of TCM monomers to precisely regulate key nodes of the NF-κB pathway.

TCM compound formulations, leveraging their multi-component and multi-target synergistic advantages, demonstrate comprehensive effects in regulating the NF-κB pathway for CRC treatment: Xiaochaihu Decoction inhibits the NF-κB/NLRP3 pathway and reduces TNF-α levels, delaying tumor progression (Shao et al., 2020); Yiyi Fuzi Baijiang Powder reduces IκBα phosphorylation and p65 nuclear translocation in the AOM/DSS model, modulating the balance of inflammatory factors (Yang et al., 2023); Banxia Xiexin Decoction induces apoptosis and suppresses EMT via the PARG/PARP1/NF-κB pathway (Huang et al., 2025); Gegen Qinlian Decoction and Changweiqing Formula inhibit IκBα phosphorylation and IKK activation, respectively, suppressing the NF-κB pathway and downstream pro-inflammatory factors (Xu et al., 2020; Wan et al., 2019). These studies on TCM compounds reflect the unique advantages of TCM in holistically regulating the NF-κB signaling network and reversing malignant tumor phenotypes, show as Figure 1.

## 4 TCM as a “signaling network inhibitor”: a SWOT analysis and future perspectives

### 4.1 Strengths: multi-target synergy and holistic regulation

The core strength of TCM lies in its inherent capacity for multi-target, multi-pathway network regulation, which aligns with the complexity of CRC signaling networks. Unlike single-target inhibitors that often face resistance due to pathway redundancy and compensatory activation, TCM formulations can simultaneously modulate key nodes across interconnected pathways (e.g., PI3K/Akt, Wnt/β-catenin, NF-κB) (Sun et al., 2024; Zheng et al., 2025; Wang et al., 2022). This system-level intervention may disrupt the tumor’s adaptive signaling network more effectively, potentially delaying or preventing drug resistance. Furthermore, TCM’s holistic approach extends beyond direct tumor cytotoxicity to encompass modulation of the tumor immune microenvironment, mitigation of chronic inflammation, and improvement of host systemic balance (e.g., alleviating chemotherapy-induced side effects, enhancing physical fitness) (Rao et al., 2025; Zheng et al., 2025). This aligns with the concept of TCM as a complementary medicine that supports the host while attacking the disease, as illustrated in studies combining natural compounds with conventional therapies to mitigate adverse effects and improve outcomes (Gao et al., 2025; Hu et al., 2016).

### 4.2 Weaknesses: challenges in standardization, material definition, and translational validation

A fundamental weakness hindering the objective evaluation and reproducibility of TCM research lies in the inadequate and inconsistent definition of the investigated materials (Heinrich et al., 2022). While this review catalogs numerous active components and formulas, the underlying studies frequently lack the pharmacological rigor demanded for modern drug development. Critical details are often missing or underreported:

For Herbal Extracts and Compounds: Many studies omit the full botanical nomenclature (genus, species, authority), the precise plant part used (e.g., root, leaf), the extraction protocol (solvent, temperature, duration), and quantitative standardization data (e.g., HPLC fingerprint, content of a marker compound). The simple label “XX extract” is pharmacologically insufficient (Upton et al., 2024).

For TCM Compound Formulas: The description is often limited to the formula name (e.g., “Zuojin Pill”). Crucial information such as the specific source and quality of each constituent herb, the preparation method (decoction, pill, granule), and the chemical fingerprint or quality control standards of the final preparation are rarely provided. This makes it impossible to ascertain if different studies investigating the same named formula are actually testing comparable entities (Qiu et al., 2016).

Gap Between *In Vitro* and *In Vivo* Feasibility: As previously noted in Section 5.3, the pharmacological relevance of many *in vitro* findings is questionable due to the use of concentrations unlikely to be achieved *in vivo*. This issue is exacerbated by the lack of parallel pharmacokinetic (PK) and absorption, distribution, metabolism, and excretion (ADME) studies for most TCM components, which are essential to bridge this gap ().

This deficiency in material definition not only challenges the interpretation and comparison of individual studies but also represents a major barrier to the global acceptance and integration of TCM into evidence-based oncology (Zhang J. et al., 2024). High-quality research must begin with well-characterized, reproducible test materials.

### 4.3 Opportunities: integration with modern science and precision medicine

The convergence of TCM with contemporary biomedical technologies presents unprecedented opportunities. Network pharmacology and multi-omics technologies (genomics, proteomics, metabolomics) offer powerful tools to deconvolute the “component-target-pathway-disease” networks of TCM, moving its study from empirical description to mechanistic elucidation (Zhang et al., 2019; Li and Zhang, 2013). Inspired by research on molecular biomarker panels for cancer screening, a key opportunity lies in identifying TCM-specific or therapy-response predictive biomarkers (Hajibabaie et al., 2025). This could enable precision patient stratification by integrating TCM syndrome differentiation with molecular subtyping, thereby identifying individuals most likely to benefit from specific TCM network interventions. Furthermore, innovative clinical trial designs adapted to evaluate “multi-target network regulation” and strategic combinations of TCM with chemotherapy, targeted therapy, or immunotherapy (as seen in studies combining natural agents with 5-FU) represent promising avenues to demonstrate synergistic efficacy and reduced toxicity (Abedpoor et al., 2024; Zhang D. et al., 2024).

### 4.4 Threats: scientific scrutiny, regulatory hurdles, and knowledge gaps

The path forward is not without threats. The lack of universally accepted standardization and mechanistic clarity can fuel scientific skepticism, hindering global acceptance (Normile, 2003). Regulatory frameworks in many regions are not fully equipped to evaluate complex multi-component therapies, creating significant barriers to market approval (Qiu, 2007). Additionally, there is a translational knowledge gap concerning the intricate interplay between TCM components, their metabolism, the gut microbiome, and the host immune system in the context of CRC (Bu et al., 2023). Without addressing these gaps through interdisciplinary research, the full potential of TCM as a sophisticated “signaling network inhibitor” may remain unrealized.

### 4.5 Future perspectives: strategic integration for next-generation oncology

To translate the “signaling network inhibitor” paradigm into clinical reality, a strategic, multi-pronged research agenda is essential. Future efforts must prioritize: 1) Advanced phytochemistry and pharmacokinetics to define bioactive constituents and their attainable *in vivo* levels; 2) Mechanistic deconvolution using systems biology to validate network-level effects; 3) Development of biomarker-guided precision TCM to personalize interventions; and 4) Conduct of high-quality pragmatic clinical trials that evaluate TCM as an integrated component of comprehensive CRC management, focusing on hard endpoints like survival, quality of life, and reduction of treatment-related morbidity (Zou et al., 2024; Xing et al., 2023; Yuen et al., 2022). By embracing these directions, TCM can evolve from an adjunctive therapy to a cornerstone of innovative, integrative oncology strategies aimed at overcoming drug resistance and improving patient survivorship.

## 5 Summary and outlook

### 5.1 The unique advantages of traditional Chinese medicine as a “signaling network inhibitor”

Compared to modern chemical or biological inhibitors characterized by high selectivity and single-target action, TCM demonstrates distinct paradigmatic advantages in intervening in complex disease signaling networks (Hopkins, 2008).

First, multi-target synergy and network regulation represent its core strength. While existing inhibitors typically target a single abnormal protein (e.g., kinases) with precision and efficiency, tumor cells can rapidly develop resistance through bypass activation or compensatory mechanisms. In contrast, TCM formulas or active component groups can simultaneously act on multiple key pathway nodes (such as PI3K/Akt, Wnt/β-catenin, etc.) and their interfaces, weakening the overall functionality and robustness of the network at a systemic level. This may more effectively prevent or delay the emergence of drug resistance (Sun et al., 2024; Wang et al., 2022). Second, systemic regulation and holistic homeostasis restoration are notable features. Conventional inhibitors often focus directly on killing tumor cells or blocking a single proliferation signal, whereas TCM, while inhibiting abnormal proliferation, frequently exhibits multidimensional effects such as modulating the tumor immune microenvironment, suppressing inflammation, influencing cellular metabolism, and inducing differentiation (Zheng et al., 2025; Gao et al., 2025). This aligns more closely with TCM’s holistic treatment philosophy of “strengthening the body’s resistance and eliminating pathogenic factors” to restore internal environmental balance. Third, TCM exhibits a “mild” mode of action and potentially lower toxicity. The synergistic effects of multiple TCM components may produce cumulative outcomes through moderate regulation of multiple targets, rather than extreme inhibition of a single pathway. This could ensure therapeutic efficacy while reducing off-target toxic side effects caused by excessive inhibition of specific targets (Wagner, 2011).

Despite challenges in component clarity, pharmacokinetics, and direct potency, TCM’s “multi-target, low-affinity, systemic regulation” mode of action provides a complementary solution distinct from the traditional “single-target sniping” strategy. It addresses complex biological issues such as tumor heterogeneity, signaling redundancy, and microenvironment interactions. Viewing TCM as a “signaling network inhibitor” offers a theoretical summarization of this systemic pharmacological advantage and opens new avenues for developing next-generation integrated anticancer strategies (Li and Weng, 2017).

### 5.2 Current clinical application status and challenges

In clinical practice, Chinese herbal monomers and compound formulas primarily play an auxiliary and comprehensive management role in the treatment of colorectal cancer. Their application is mainly reflected in two aspects: First, classical compound formulas based on syndrome differentiation and treatment—such as those for fortifying the spleen, clearing heat, or promoting blood circulation—are used to alleviate the toxic side effects of chemotherapy and radiotherapy, modulate immunity, and improve patients’ quality of life (Chen et al., 2023). Second, certain modern-processed Chinese medicine injections or oral formulations—such as Cinobufacini, Kanglaite, and Shenyi Capsules—are combined with chemotherapy or targeted therapy, showing potential value in enhancing efficacy and reducing toxicity in clinical studies (Qi et al., 2010). However, current applications largely rely on expert experience and traditional theories. There is generally a lack of support from large-scale, high-quality randomized controlled trial evidence, and the standardization of formulations and the clarity of active components remain critical challenges for achieving broad recognition and precise integration (Efferth and Koch, 2011).

### 5.3 In-depth analysis of core translational challenges

This review cites extensive *in vitro* cell-based evidence, where the reported effective concentrations of TCM monomers (typically in the micromolar range, e.g., 95 μmol/L for DHA) serve as an important foundation for their pharmacological activity. However, we must clearly recognize that there is often a significant gap between these *in vitro* effective concentrations and the currently known achievable plasma concentrations of these compounds in the human body. For instance, the peak blood concentrations of artemisinin compounds in clinical antimalarial treatment are typically in the nanomolar range, far below the concentrations required in many *in vitro* anticancer studies. This discrepancy represents a core challenge in translational medicine (Uysal et al., 2019), (Balunas and Kinghorn, 2005).

The potential reasons for this gap are complex and multifaceted. First, orally administered TCM formulas may achieve relatively high local concentrations in the gastrointestinal tract—particularly in the target organ of colorectal cancer—which differs from systemic pharmacokinetics (Li et al., 2023). Second, many TCM components undergo extensive metabolic transformation *in vivo*, and the potency of their active metabolites may far exceed that of the parent compounds (Li et al., 2022). Furthermore, the core advantage of TCM’s “multi-component, multi-target” approach implies that low concentrations of individual components, through synergistic or sequential actions, may produce network effects equivalent to or even stronger than a high-concentration impact from a single compound—a phenomenon difficult to replicate in simplified models (Zhang J. et al., 2024).

Therefore, the primary value of current *in vitro* studies lies in revealing mechanisms of action and target possibilities, thereby mapping a “biological roadmap” for subsequent research. Future research must focus on bridging this “concentration gap.” This requires structural optimization, the development of novel drug delivery systems (such as nano-targeted delivery), and the rational design of compound formulas based on synergy principles. The ultimate goal is to achieve and maintain a steady-state concentration of active components within the safety window at the target tissue, sufficient to modulate the signaling network. Acknowledging and systematically investigating this gap is a critical step in transforming TCM from an excellent “concept of signaling network modulation” into a verifiable “clinical network therapy”.

### 5.4 Future translational directions and strategies

The core contribution of this review lies in systematically proposing and substantiating, for the first time, the innovative paradigm of viewing TCM as a “signaling network inhibitor.” This perspective transcends the dominant single-target mindset in conventional cancer therapy, elevating TCM’s holistic action characteristics—its “multi-component, multi-target, multi-pathway” approach—to the theoretical level of systemic intervention against disease signaling networks. This paradigm not only provides a unified framework for explaining TCM’s synergistic effects in regulating complex pathways such as PI3K/Akt/mTOR, MAPK, Wnt/β-catenin, and their crosstalk but also fundamentally elucidates its unique advantages in addressing tumor heterogeneity, blocking compensatory activation, and reversing clinical drug resistance (Zheng et al., 2025; Yang and Lao, 2019).

Based on the theoretical framework of the “signaling network inhibitor,” the value of TCM in colorectal cancer treatment is no longer confined to the discovery of single active components or the validation of specific pathways. Instead, it offers a novel integrated therapeutic philosophy. It suggests that future anti-cancer strategies should shift from “precision strikes” to “network regulation,” aiming to reshape the homeostasis of the tumor system through multi-target, multi-layered, and moderate interventions. This approach may yield more durable therapeutic effects with a lower likelihood of inducing resistance. This concept lays a critical theoretical foundation for developing next-generation integrated anti-cancer strategies that combine Chinese and Western medicine.

Looking ahead, advancing the translational application of this paradigm urgently requires the deep integration of TCM’s holistic perspective with modern systems biology methods. By combining network pharmacology, multi-omics analysis, and artificial intelligence, it will be possible to gradually decipher the material basis and dynamic mechanisms through which TCM formulas achieve “network inhibition.” Concurrently, designing matched clinical trials and exploring precise patient stratification based on biomarkers and TCM syndromes will be the core pathway for advancing TCM from a theoretical paradigm to clinical practice, thereby realizing its modernization and international value (Li and Zhang, 2013; Yuen et al., 2022).
